# Novel Electrochemical Sensors Based on Cuprous Oxide-Electrochemically Reduced Graphene Oxide Nanocomposites Modified Electrode toward Sensitive Detection of Sunset Yellow

**DOI:** 10.3390/molecules23092130

**Published:** 2018-08-24

**Authors:** Quanguo He, Jun Liu, Xiaopeng Liu, Yonghui Xia, Guangli Li, Peihong Deng, Dongchu Chen

**Affiliations:** 1 School of Materials Science and Energy Engineering, Foshan University, Foshan 528000, China; hequanguo@126.com (Q.H.); liu.jun.1015@163.com (J.L.); 2 College of Life Sciences and Chemistry, Hunan University of Technology, Zhuzhou 412007, China; amituo321@163.com; 3 Zhuzhou Institute for Food and Drug Control, Zhuzhou 412000, China; sunnyxia0710@163.com; 4 Department of Chemistry and Material Science, Hengyang Normal University, Hengyang 421008, China; dph1975@163.com

**Keywords:** cuprous oxide nanoparticles, reduced graphene oxide, modified electrode, sunset yellow, second-derivative linear sweep voltammetry

## Abstract

Control and detection of sunset yellow is an utmost demanding issue, due to the presence of potential risks for human health if excessively consumed or added. Herein, cuprous oxide-electrochemically reduced graphene nanocomposite modified glassy carbon electrode (Cu_2_O-ErGO/GCE) was developed for the determination of sunset yellow. The Cu_2_O-ErGO/GCE was fabricated by drop-casting Cu_2_O-GO dispersion on the GCE surface following a potentiostatic reduction of graphene oxide (GO). Scanning electron microscope and X-ray powder diffractometer was used to characterize the morphology and microstructure of the modification materials, such as Cu_2_O nanoparticles and Cu_2_O-ErGO nanocomposites. The electrochemical behavior of sunset yellow on the bare GCE, ErGO/GCE, and Cu_2_O-ErGO/GCE were investigated by cyclic voltammetry and second-derivative linear sweep voltammetry, respectively. The analytical parameters (including pH value, sweep rate, and accumulation parameters) were explored systematically. The results show that the anodic peak currents of Cu_2_O-ErGO /GCE are 25-fold higher than that of the bare GCE, due to the synergistic enhancement effect between Cu_2_O nanoparticles and ErGO sheets. Under the optimum detection conditions, the anodic peak currents are well linear to the concentrations of sunset yellow, ranging from 2.0 × 10^−8^ mol/L to 2.0 × 10^−5^ mol/L and from 2.0 × 10^−5^ mol/L to 1.0 × 10^−4^ mol/L with a low limit of detection (S/N = 3, 6.0 × 10^−9^ mol/L). Moreover, Cu_2_O-ErGO/GCE was successfully used for the determination of sunset yellow in beverages and food with good recovery. This proposed Cu_2_O-ErGO/GCE has an attractive prospect applications on the determination of sunset yellow in diverse real samples.

## 1. Introduction

Sunset yellow, as a common azo colorant, has been widely added in several beverages (such as carbonated beverage, orange juice, and Fanta drink) and food (i.e. candies, cakes, cheese) to improve the appearance and appetite [1,2]. Sunset yellow contains an azo group (-N=N-) and aromatic ring structure, which may cause mutagenic and carcinogenic risk for human [3,4]. As a result, it can bring many serious health problems, such as hepatocellular damage, kidney failures, headache, cancers, and attention deficit hyperactivity disorder (ADHD), when it is excessively consumed [5]. Hence, the additive amount of sunset yellow in beverages and food demands strict control and regulation. European Food Safety Authority (EFSA) recommends an acceptable daily intake (ADI) for sunset yellow of 1.0 mg kg^−1^ body weight [6]. In some countries, it was explicitly stated that the permitted maximum content of sunset yellow in nonalcoholic beverages is 100 μg mL^−1^ [7]. Moreover, Finland and Norway even has already banned the use of sunset yellow in foods [6]. While considering the food quality and safety, it is urgent to develop reliable analytical techniques for the quick detection of this food dye. 

At present, various techniques have been developed for sunset yellow, including high performance liquid chromatography (HPLC) [8], thin layer chromatography [9], spectrophotometry [10], fluorometry [11], and capillary electrophoresis [12]. Although these methods have been proved to be reliable, they still have some disadvantages, such as time-consuming, complicated preprocessing, and expensive equipment. Recently, electrochemical analysis has been widely used for the detection of bioactive molecules, nutrients, food additives, as well as contaminants, due to its considerable merits such as low cost, simple operation, rapid response, high sensitivity and sensitivity. As we all know, the key issue for electrochemical detection toward sunset yellow is to develop ultrasensitive modified electrodes.

Nanostructure precious metals or alloys have become the preferred electrode modification materials for the sensitive detection of sunset yellow, due to their superior electrocatalytic activity [7,13,14,15,16,17]. For example, Wang and coworkers developed a promising electrochemical sensor based on Au-Pd and reduced graphene oxide nanocomposite decorated electrode (Au-Pd-RGO/GCE) [17]. The Au-Pd-RGO/GCE exhibited good stability, superior electrocatalytic performance, low detection limit (1.5 nmol/L), and wide response range (0.686–331.686 μmol/L). Pd-Ru nanoparticles incorporated carbon aerogel nanocomposites (Pd-Ru/CA) have been successfully used for electrochemical detection and catalytic degradation of sunset yellow [16]. The Pd-Ru/CA nanocomposite decorated screen printed carbon electrode (Pd-Ru/CA/SPCE) showed a low detection limit (7.1 nmol/L) and high sensitivity (3.571 μA/(μmol/L cm^2^). Electrochemical sensing platform based on Au NPs and reduced graphene modified GCE (Au NPs/RGO/GCE) was constructed for the quantitative analysis of sunset yellow, and the Au NPs/RGO/GCE had excellent catalytic activity toward the oxidation of sunset yellow [15]. The proposed electrode showed wide linear response range of 0.002–109.14 μmol/L and low detection limit of 2 nmo/L (S/N = 3). Au NPs decorated carbon-paste electrode (Au NPs/CPE) was also fabricated for the simultaneous detection sunset yellow and Tartrazine [7]. The Au NPs/CPE displayed low detection limits of 3.0 × 10^−8^ and 2.0 × 10^−9^ mol/L for sunset yellow and Tartrazine, respectively. These precious metals-based modified electrodes can detect sunset yellow at the nanomole level; however, the scarcity and high cost of precious metals or alloys have seriously hindered the broad practical applications.

When compared to precious metals or alloys, transition metals and metal oxides have outstanding advantages in terms of abundance and cost. What is more, transition metals and metal oxides also have excellent catalytic activity. In our previous work, Cu_2_O-RGO nanocomposite [18], NH_2_-Fe_3_O_4_-RGO nanocomposite [19], MnO_2_-RGO nanocomposite [20], TiO_2_-RGO nanocomposite [21], and α-MnO_2_/N-doped ketjenblack carbon composite [22,23] demonstrated excellent electrochemical sensing performance toward the detection of dopamine and Tartrazine and superior electrocatalytic activity in oxygen reduction reaction (ORR) & oxygen evolution reaction (OER). Recently, few researchers have devoted to developing transition metal oxide modified electrodes for the determination of sunset yellow. For example, Dorraji et al. [24] developed ZnO/cysteic acid nanocomposite modified electrode (ZnO/CA/GCE), and successfully used for simultaneous determination of sunset yellow and Tartrazine. The ZnO/CA/GCE exhibited two linear response ranges in the concentration ranges of 0.1–3.0 μmol/L, and 0.07–1.86 μmol/L, and detection limits of 0.03 μmol/L and 0.01 μmol/L for sunset yellow and Tartrazine, respectively. However, the linear dynamic response range is limited for trace detection of sunset yellow. The detection capacity of sunset yellow has been improved with graphene and mesoporous TiO_2_ composite [25] and ZnO/RGO/ZnO@Zn [26] modified electrodes, and they showed superior sensing performance (i.e., linear ranges, detection of limit) that was comparable to precious metal modified electrode. Although some progress has been made, there are only few related reports concerning the transition metal oxide modified electrodes. Therefore, it is still worthwhile to develop novel transition metal oxide modified electrodes for sensitive detection of sunset yellow.

Among transition oxides, cuprous oxide (Cu_2_O) is an environmentally friendly p-type semiconductor material, which has been widely used in solar cells and photo catalysis [27,28], due to its unique electronic structure and excellent catalytic performances. However, its electrical conductivity is poor due to the nature of semiconductor. To resolve this problem, Cu_2_O nanoparticles are often composited or hybrided with conductive materials [29,30,31,32,33], to decrease the charge transfer resistance and eventually enhance the electrochemical performance. Graphene, as an emerging two-dimensional (2D) carbon material, has been usually used as conductive materials in modified electrodes, owing to its high specific surface area, excellent electrical conductivity, superior electrochemical performance, and fast heterogeneous electron transfer rate. It has been reported that graphene-based modified electrodes have been widely employed for the determination of azo dyes, such as sunset yellow, Tartrazine, and Amaranth [34,35,36]. However, to our best knowledge, Cu_2_O/reduced graphene oxide nanocomposite modified electrode toward sensitive detection of sunset yellow has not been reported.

Graphene usually prepared from graphene oxide (GO) by electrochemical reduction method in the field of electrochemical analysis. The chemically reduced graphene oxide is hydrophobic, due to the removal of most oxygen-containing functional groups. As a result, the chemically graphene oxide tends to agglomerate resulting in a degradation on sensing performance. The agglomeration issue can be overcomed by introducing the surfactants [37], which can effectively improve the dispensability. However, the electrical conductivity also declined due to the use of surfactants. Electrochemically reduction method is a green and efficient method to obtain reduced graphene oxide that not require any reductants. Moreover, the residual oxygen-containing functional groups can be tuned by facial adjusting the electrochemical parameter, such as reduction potential, reduction time, and scanning cycles [18,19,21]. In other words, the property of reduced graphene oxide can be tailored by electrochemical parameters. For these reasons, the electrochemically reduced graphene oxide (ErGO) have been widely used for constructing diverse sensors.

Inspired by the foregoing reports, herein ErGO was composited with low cost and excellent electrocatalytic activity Cu_2_O nanoparticles, aiming to develop a cost-effective, high sensitive, and good selective modification materials to substitute the precious metal-based materials. Meanwhile, the Cu_2_O-ErGO nanocomposites are expected to exert their synergistic sensitizing effects to improve the sensing performance. Then, Cu_2_O-ErGO nanocomposites was modified on the surface of the glassy carbon electrode (GCE) to construct a novel sensor toward sunset yellow. The Cu_2_O-ErGO modified glassy carbon electrode (Cu_2_O-ErGO/GCE) was prepared while using a facile drop-casting technique in combination with electrochemical reduction. The electrochemical behavior of sunset yellow on the Cu_2_O-ErGO/GCE were investigated by cyclic voltammetry and second-derivative linear sweep voltammetry. The effect of detection conditions (such as pH value, sweep rate, and accumulation parameters) on the electrochemical response were also explored. Finally, the proposed Cu_2_O-ErGO/GCE was used to detect the content of sunset yellow in soda drinks, orange juice, and candies samples while using second-derivative linear sweep voltammetry.

## 2. Results and Discussion

### 2.1. Morphology and Microstructural Characterization

**Figure 1:**
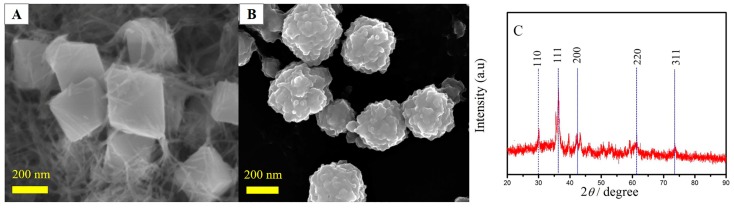
The scanning electron microscope (SEM) photos of cuprous oxide (Cu_2_O) nanoparticles (**A**) and cuprous oxide-electrochemically reduced graphene oxide (Cu_2_O-ErGO) nanocomposites (**B**); and, (**C**) The X-ray diffraction (XRD) pattern of Cu_2_O nanoparticles.

The surface morphologies of Cu_2_O nanoparticles (Cu_2_O NPs) and Cu_2_O-ErGO nanocomposites were characterized by scanning electron microscope (SEM, Hitachi S-3000N, Tokyo, Japan). The SEM images are shown in Figure 1A,B, respectively. The Cu_2_O NPs exhibit cubic-like structure with uniform size, and the particle size is estimated to about 150 nm. Obviously, the thin layer ErGO sheets were successfully coated on the surface of Cu_2_O nanoparticles. Moreover, the particle size of Cu_2_O-ErGO nanocomposite increases slightly, which facilitates the adsorption of sunset yellow. The Cu_2_O NPs were further characterized by X-ray diffraction (XRD, JEOL JEM-2010 (HT, Tokyo, Japan) and the XRD pattern of Cu_2_O NPs is plotted in Figure 1C. The diffraction peaks of Cu_2_O nanoparticles are clearly indexed into the pure cubic phase of Cu_2_O (JSPDS78-2076), suggesting that the cubic phase of Cu_2_O nanoparticles was prepared.

### 2.2. Electrochemical Behavior of Sunset Yellow on Modified Electrodes

**Figure 2:**
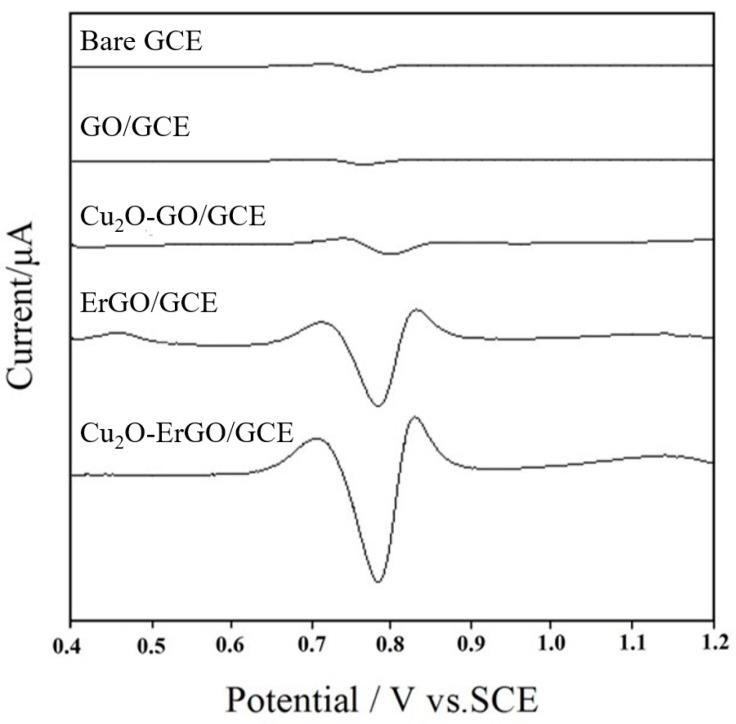
Second-derivative linear sweep voltammograms of 1.0 × 10^−5^ mol/L sunset yellow on the different electrodes.

Second-derivative linear sweep voltammetric responses of 1.0 × 10^−5^ mol/L sunset yellow on different electrodes are presented in Figure 2. On the bare GCE, a weak anodic peak of sunset yellow appears at 798 mV with the anodic peak current (*i*_pa_) of 0.725 μA. On the GO/GCE, the oxidation peak current of sunset yellow decreases to 0.497 μA, which is mainly due to the present of the poor electrical conductivity of GO. On the Cu_2_O-GO/GCE, an apparent anodic peak of occurred at 770 mV, and the *i*_pa_ increases to 0.925 μA, probably owing to the electrocatalytic activity of Cu_2_O nanoparticles. When the GO was electrochemically reduced to ErGO, the anodic peak appears at 792 mV and the *i*_pa_ increases to 16.93 μA. This phenomenon may be related to the high electrical conductivity, large specific surface area, and rapid heterogeneous electron transfer rate of ErGO. Moreover, the adsorption capacity of sunset yellow on the electrode surface is improved greatly by the π-π interaction, because the conductive carbon-conjugated networks are restored after the reduction process. When the GCE was modified with Cu_2_O-ErGO nanocomposites, the *i*_pa_ is the largest (18.08 μA), which is about 25 fold greater than that of bare GCE. It mainly due to the synergistic enhancement effect between Cu_2_O nanoparticles and ErGO sheets, which significantly improves the sensitivity of sunset yellow detection.

**Figure 3:**
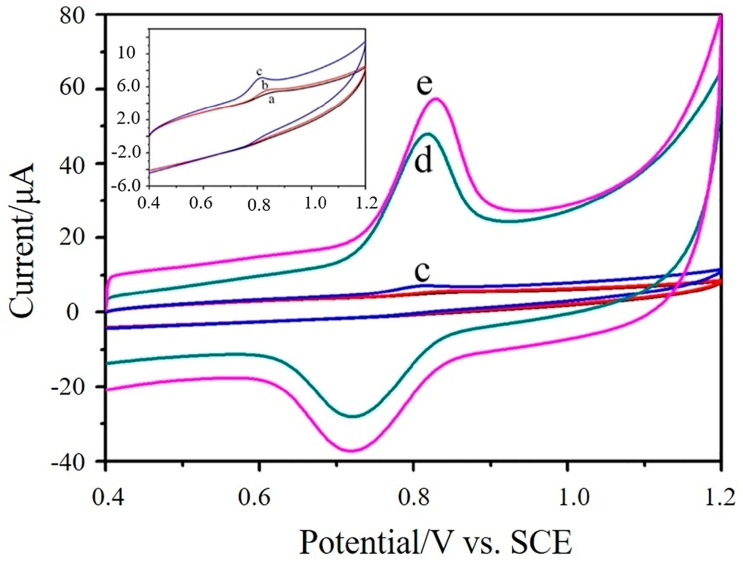
Cyclic voltammograms of 1.0 × 10^−5^ mol/L sunset yellow recorded on the glassy carbon electrode (GCE) (a), graphene oxide/glassy carbon electrode (GO/GCE) (b), Cu_2_O-graphene oxide nanocomposite modified glass carbon electrode (Cu_2_O-GO/GCE) (c), electrochemically reduced graphene oxide modified glass carbon electrode (ErGO/GCE) (d) and Cu_2_O-ErGO modified glassy carbon electrode (Cu_2_O-ErGO/GCE) (e). The inset is the magnification of the cyclic voltammograms recorded on the GCE (a), GO/GCE (b) and Cu_2_O-GO/GCE (c).

The electrochemical behavior of 1.0 × 10^−5^ mol/L sunset yellow on the GCE (a), GO/GCE (b), Cu_2_O-GO/GCE (c), ErGO/GCE (d), and Cu_2_O-ErGO/GCE (e) were further investigated by cyclic voltammetry (Figure 3). All of the electrodes appear a pair of redox peaks, meaning that sunset yellow undergoes a quasi-reversible process. Obviously, a pair of sharp redox peaks occurs on the ErGO/GCE and Cu_2_O-ErGO/GCE. Furthermore, the order of anodic peak currents obtained from cyclic voltammograms is consistent with the second-derivative linear sweep voltammograms, which further confirms that Cu_2_O-ErGO nanocomposites can significantly enhance the electrochemical response toward sunset yellow. 

### 2.3. Effect of pH Value

**Figure 4:**
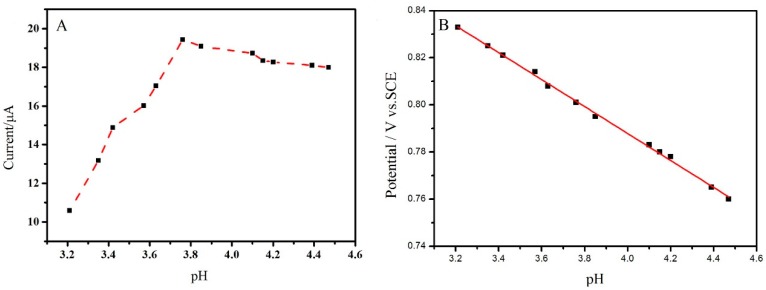
(**A**) Effects of pH value on the anodic peak currents of 1.0 × 10^−5^ mol/L sunset yellow on the Cu_2_O-ErGO/GCE; and, (**B**) The plot of the anodic peak potential of 1.0 × 10^−5^ mol/L sunset yellow against pH value.

Since proton (H^+^) plays an important role on the redox of sunset yellow, so it is worthwhile investigating the influence of pH value on the response peak current of sunset yellow. The *i*_pa_ of sunset yellow recorded in various pH PBS solution are depicted in Figure 4A. The *i*_pa_ of sunset yellow increases gradually as the pH value increases. When the pH value increases to 3.8, the largest *i*_pa_ is obtained. Afterwards the *i*_pa_ decreases slowly with the pH value further increasing. Hence, the pH 3.8 PBS solution was employed as supporting electrolytes on the subsequent experiments. Furthermore, the anodic peak potential (*E*_pa_) of sunset yellow is negatively shifted with the increase of pH value. As plotted in Figure 4B, there is a good linear relationship between *E*_pa_ and pH value, confirming that protons are involved in the oxidation of sunset yellow. The linear regression equation can expressed as *E*_pa_ (V) = −0.0570 pH + 1.0167 (R^2^ = 0.998). According to Nernst equation, its slope (−0.0570 V/pH) approaches to the theoretical value (−0.0590 V/pH), suggesting that the equal amounts of proton and electron involve in the electrochemical oxidation of sunset yellow [38].

### 2.4. Effect of Sweep Rates

**Figure 5:**
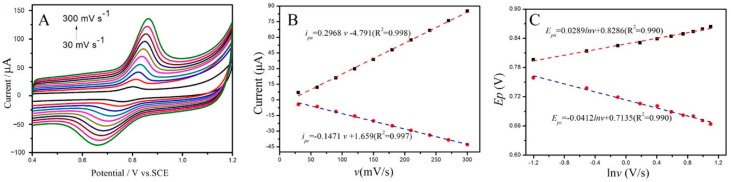
(**A**) Cyclic voltammograms of 1.0 × 10^−5^ mol/L sunset yellow on the Cu_2_O-ErGO/GCE recorded at various sweep rates (30 mV/s–300 mV/s); (**B**) Linear plots of anodic and cathodic peak currents (*i*_pa_ and *i*_pc_) of sunset yellow against sweep rates (*v*); and (**C**) Linear plots of andic and cathodic peak potential (*E*_pa_ and *E*_pc_) of sunset yellow against Napierian Logarithm of sweep rates (ln*v*). Supporting electrolytes: 0.1 mol/L PBS.

Sweep rate is a crucial parameter that directly affects the electrochemical response of analysts on the modified electrodes. Moreover, it is a powerful tool to reveal the electrochemical reaction mechanism. Cyclic voltammograms at various sweep rates were recorded at 0.1 mol/L PBS solution containing 1 × 10^−5^ mol/L sunset yellow while using the Cu_2_O-ErGO/GCE, and their corresponding cyclic voltammograms are shown in the Figure 5A. As expected, with sweep rates increasing, the anodic peaks shift toward positive direction, while the cathodic peaks shift negatively, indicating that the oxidation of sunset yellow on the Cu_2_O-ErGO/GCE is quasi-reversible. Both the anodic peak currents (*i*_pa_) and cathodic peak currents (*i*_pc_) increase with the sweep speeding up, however, the background currents also increase. To purist high signal-to-noise (S/N), a suitable sweep rate is recommended as 100 mV/s. It can be clearly seen from Figure 5B that both the anodic peak currents (*i*_pa_) and cathodic peak currents (*i*_pc_) of sunset yellow is nearly linear with the sweep rates (*v*). Their linear equations are expressed as: *i*_pa_ (μA) = 0.2968 *v* (mV/s) − 4.791 (R^2^ = 0.998) and *i*_pc_ (μA) = −0.1471 *v* (mV/s) + 1.659 (R^2^ = 0.997), suggesting that the oxidation of sunset yellow on the Cu_2_O-ErGO/GCE is controlled by the adsorption process [39].

**Figure 6:**
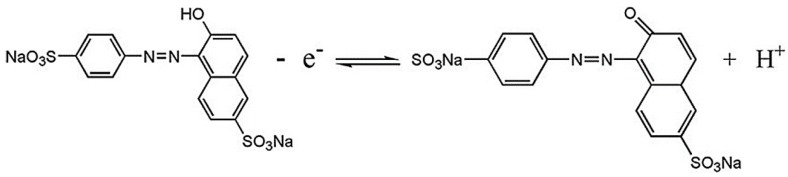
The mechanism for the electrochemical processes of sunset yellow on the Cu_2_O-ErGO/GCE.

It is observed that both the anodic peak potential (*E*_pa_) and the cathodic peak potential (*E*_pc_) is well linear to the Napierian Logarithm of sweep rates (ln*v*). Their corresponding linear equations are *E*_pa_ (V) = 0.0289ln*v* (V/s) + 0.8286 (R^2^ = 0.990) and *E*_pc_ (V) = − 0.0412ln*v* (V/s) + 0.7135 (R^2^ = 0.990). As for an adsorption-controlled and quasi-reversible process, according to the Lavrion equation [40], the peak potential and the sweep rate follows the following relationship:

(1) $$E_{\mathrm{pa}}=E^{0^{\prime }}+\frac{RT}{(1-\alpha )nF}[0.78+\ln(\frac{D^{1/2}}{K_{s}})-\frac{1}{2}\ln\frac{RT}{(1-\alpha )nF}]+\frac{RT}{2(1-\alpha )nF}\ln v$$

(2) $$E_{\mathrm{pc}}=E^{0^{\prime }}+\frac{RT}{\alpha nF}[0.78+\ln(\frac{D^{1/2}}{K_{s}})-\frac{1}{2}\ln\frac{RT}{\alpha nF}]-\frac{RT}{2\alpha nF}\ln v$$

where *E*_pa_ (V) and *E*_pc_ (V) represents the anodic peak potential and the cathodic peak potential, respectively; *v* (V/s) denotes the sweep rate; α is the charge transfer coefficient; *k*_s_ is the heterogeneous electron transfer rate; *n* is the electron transferred number; *T* is Kelvin temperature; *F* is Faraday constant (96,480 C/mol); and, *R* is molar gas constant (8.314 J/(mol·K)). Combining the slopes of Equations (1) and (2) with the *E_pa_*/*E_pc_* vs. *lnv* equations, the charge transfer coefficient α is estimated to be 0.45 and the electron transferred number n is around 1. Since the equal amount of proton and electron participates in the oxidation process, the electrochemical oxidation of sunset yellow is 1 electron and 1 proton process, which is in accordance with the previous studies [39,41]. Hence, the electrochemical oxidation mechanism of sunset yellow on the Cu_2_O-ErGO/GCE can be inferred in Figure 6.

### 2.5. Effect of Acumualtion Parameters

**Figure 7:**
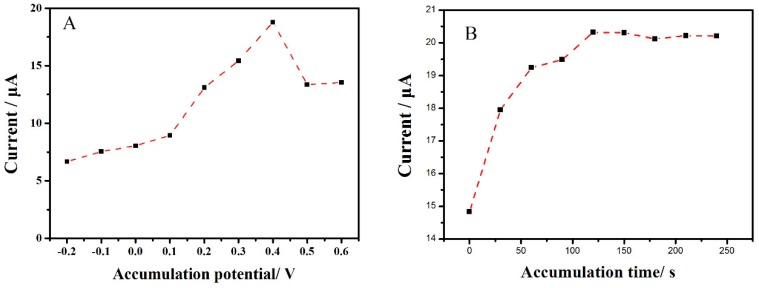
Influences of accumulation potential (**A**), and accumulation time (**B**) on the anodic peak currents of 1.0 × 10^−5^ mol/L sunset yellow on the Cu_2_O-ErGO/GCE.

Accumulation is a simple and effective technique to improve the electrochemical response. Since the electrochemical oxidation of sunset yellow is an adsorption-controlled process, so accumulation were performed before second-derivative linear sweep voltammetry. As we all know, accumulation potential as well as time are two important parameters that affect the response peak current greatly, so it is a worthwhile optimization. The Cu_2_O-ErGO/GCE was accumulated at different accumulation potential for 240 s firstly. Then, their anodic peak currents (*i*_pa_) of sunset yellow were recorded in 0.1 mol/L PBS solution (pH 3.8) while using second-derivative linear sweep voltammetry. The effect of the accumulation potential on the *i*_pa_ of sunset yellow is presented in Figure 7A. The *i*_pa_ increases gradually with the rising of accumulation potential. When the accumulation potential reaches 0.4 V, the strongest *i*_pa_ is obtained. Afterwards, the *i*_pa_ decreases with the accumulation potential further increasing. Therefore, 0.4 V was selected in the subsequent experiments. Furthermore, the influence of accumulation time was also explored. Similarly, the Cu_2_O-ErGO/GCE was accumulated at an optimized accumulation potential for various time. Then, their *i*_pa_ of sunset yellow were recorded and compared. As shown in Figure 7B, the *i*_pa_ increases with the prolong of the accumulation during the first 180 s; then *i*_pa_ keep stable with the accumulation time further prolonging, demonstrating that the adsorption of sunset yellow achieved saturated. Hence, 180 s is recommended as the optimum accumulation time. 

### 2.6. Standard Curves, Linear Range and Limit of Detection 

**Figure 8:**
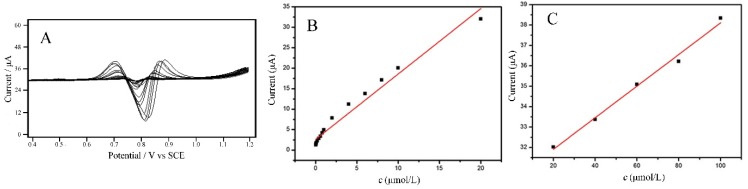
(**A**) Second-derivative linear sweep voltammograms of sunset yellow with various concentrations on the Cu_2_O-ErGO/GCE; Calibration curves between the anodic peak current and the concentrations of sunset yellow ranging from 2.0 × 10^−8^ to 2.0 × 10^−5^ mol/L (**B**) and from 2.0 × 10^−5^ to 1.0 × 10^−4^ mol/L(**C**).

With the optimal analytical parameters, the *i*_pa_ of different concentrations of sunset yellow standard solution was determined by second-derivative linear sweep voltammetry. Figure 8A shows the second-derivative linear sweep voltammograms of various concentrations of sunset yellow. There are two linear response ranges for the detection of sunset yellow, namely 2.0 × 10^−8^ ~ 2.0 × 10^−5^ mol/L (Figure 8B) and 2.0 × 10^−5^ mol/L ~ 1.0 × 10^−4^ mol/L (Figure 8C). Their corresponding linear equations are *i*_pa_ (μA) = 1.597c (μmol/L) + 2.628 (R^2^ = 0.973) and *i*_pa_ (μA) = 0.0775c (μmol/L) + 30.36 (R^2^ = 0.992), respectively. The limit of detection (LOD, S/N = 3) is estimated to be 6.0 × 10^−9^ mol/L. The linear response range is lower than the permitted maximum content of sunset yellow (2.2 × 10^−4^ mol/L), so that the Cu_2_O-ErGO/GCE can be applied to detection sunset yellow by a direct or the dilution method. A comparison on sensing performances toward sunset yellow between the existing modified electrodes and Cu_2_O-ErGO/GCE is summarized on Table 1.

The linear ranges and LOD of the proposed Cu_2_O-ErGO/GCE are at least comparable to and even better than most of the previous reports. Moreover, Cu_2_O-ErGO/GCE have outstanding advantages over noble metal modified electrodes (such as Au NPs/CPE [7], Au-Pd-RGO/GCE [17], CTAB-Gr-Pt/GCE [44], GO/AgNPs-MIPs/GCE [45] and PDDA-Gr-Pd/GCE [47] in terms of the cost and electrode fabrication.

### 2.7. Interference and Reproducibility Investigation

**Figure 9:**
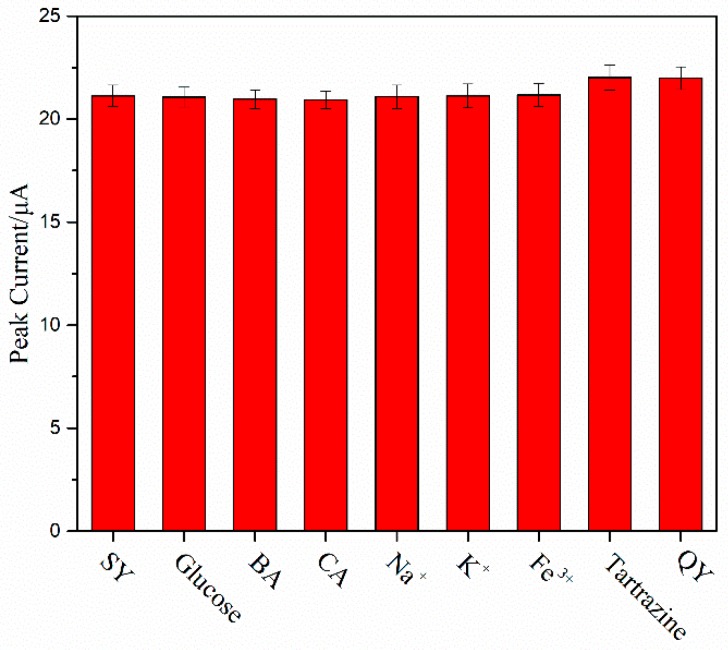
The response peak current of Cu_2_O-ErGO/GCE for 1 × 10^−5^ mol/L sunset yellow (SY) in the presence of interfering substances (*n* = 3). The concentration of glucose, benzoic acid (BA), citric acid (CA), Na^+^, K^+^, Fe^3+^, were 1 × 10^−3^ mol/L. The concentrations of Tartrazine, quinoline yellow (QY) were 1 × 10^−4^ mol/L.

Prior to the detection of real samples, the anti-interference and reproducibility was also investigated to validate the practicability of the proposed Cu_2_O-ErGO/GCE. The response peak current of pure sunset yellow solution and potential interfering compounds mixture solution were recorded and compared. It is observed that the change of *i*_pa_ of 10 μmol/L sunset yellow is less than 5% in the presence of a 100-fold concentration of glucose, benzoic acid, citric acid, Na^+^, K^+^, Fe^3+^, and 10-fold concentration of Tartrazine, quinoline yellow (Figure 9). It is demonstrating that our proposed Cu_2_O-ErGO/GCE exhibits good selectivity toward sunset yellow. The reproducibility of Cu_2_O-ErGO/GCEs were examined by continuous measurement the response peak currents of 10 μmol/L sunset yellow seven times. The result shows that the *i*_pa_ remain stable with relative standard deviation (RSD) of 2.78% (Table 2), indicating that the Cu_2_O-ErGO/GCE has good reproducibility.

### 2.8. Detection Sunset Yellow in Real Samples

Finally, Cu_2_O-ErGO/GCE was applied to determinate sunset yellow in real samples, including carbonated drinks, orange juice, and candy samples. The detect results are listed in Table 3. No anodic peak current is presented in the carbonated beverage sample near 798 mV, indicating that the concentration of sunset yellow is low than the limit of detection. The concentrations of sunset yellow in orange juice and candy samples are detected by 0.085 μmol/L and 0.162 μmol/L, respectively. The content of sunset yellow in these samples is lower than the permitted maximum content that is recommended by national standard (100 μg/mL, namely 2.2 × 10^−4^ mol/L). Then, an appropriate amount of sunset yellow standard solution was added to the above three samples. Standard addition test results suggests that the recoveries of sunset yellow were 98.75~102.0%, with the relative standard deviation being less than 2.85%, indicating that satisfactory results were obtained using the proposed Cu_2_O-ErGO/GCE. Together with cost-effective, quick response, high sensitive and good selectivity, the Cu_2_O-ErGO/GCE exhibits great prospects on the determination of sunset yellow in different real samples, including but not limiting to beverages, food, and nutrients.

## 3. Materials and Methods

### 3.1. Chemical and Solution

Graphite powder, NaNO_3_, concentrated H_2_SO_4_, NaOH, KMnO_4_, H_2_O_2_, CuSO_4_·5H_2_O, Na_2_HPO_4_, NaH_2_PO_4_, hydrochloric acid (HCl), polyvinylpyrrolidone (PVP), hydrate hydrazine (N_2_H_4_·H_2_O), and ethyl alcohol were analytic grade and supplied by Sinopharm Chemical Reagent Co., Ltd. (Shanghai, China). Sunset yellow was purchased from Aladdin (http://www.aladdin-e.com). All of these chemicals were directly used without further purification. A series of sunset yellow standard solutions were prepared by diluting appropriately the stock sunset yellow (1 × 10^−3^ mol/L). These standard solutions were kept in 4 °C refrigerator when not in use. Deionized water (18.2 MΩ) was used throughout the experiments.

### 3.2. Synthesis of Cu_2_O Nanoparticles

Cu_2_O nanoparticles were synthesized by the hydrothermal method referred to our previous work [18]. Specifically, 50 mg of CuSO_4_·5H_2_O and 24 mg of PVP were added into 10 mL deionized water and then stirred with ultrasonication for 30 min. Afterwards, 2 mL of 0.2 mol/L NaOH solution was added and stirred for 30 min at room temperature to obtain blue Cu(OH)_2_ precipitates. Subsequently, 6 μL of N_2_H_4_·H_2_O was added as reductant and then stirred for 20 min at room temperature to form a brick red suspension. The precipitate was separated by centrifugation at 5000 rpm, and washed repeatedly with deionized water and ethanol for three times, and vacuum-dried at 60 °C to obtain Cu_2_O nanoparticles.

### 3.3. Preparation Cu_2_O-GO Nanocomposite Dispersion

Graphene oxide (GO) is prepared from cheap graphite powder by a modified Hummers method according to our previous reports [18,19,21]. The as-prepared GO was dispersed in 100 mL of deionized water under ultrasonication for 2 h, and then centrifuged twice to obtain a golden yellow GO solution (1 mg/mL). 2 mg of Cu_2_O nanoparticles were added to 5 mL of the above GO solution, and then ultrasonically dispersed for 2 h to obtain a Cu_2_O-GO nanocomposite dispersion.

### 3.4. Preparation of Cu_2_O-ErGO/GCE

The glassy carbon electrode (GCE, ϕ = 3 mm) was polished to a form mirror-like surface with 0.05 μm alumina slurry. Then, the electrode was rinsed by deionized water and ethanol alternately (each for 1 min), and then dried by ultrapure N_2_ gas. Firstly, 5 μL of Cu_2_O-GO dispersion were transferred and coated on the surface of the GCE, and then dried under an infrared lamp to obtain Cu_2_O-graphene oxide nanocomposite modified glass carbon electrode (Cu_2_O-GO/GCE). Then, the GO component was electrochemically reduced by potentiostatic method. Specially, the Cu_2_O-GO/GCE was immersed into 0.1 mol/L PBS solution, and electrochemically reduced at −1.2 V for 120 s. For comparison, GO modified glass carbon electrode (GO/GCE), electrochemically reduced graphene oxide modified glass carbon electrode (ErGO/GCE) and Cu_2_O-GO nanocomposite modified glass carbon electrode (Cu_2_O-GO/GCE) were also fabricated by a similar method.

### 3.5. Electrochemical Measurements

All of the electrochemical measurements were carried out using a standard three-electrode assemble, comprising of bare or modified electrodes as working electrode, saturated calomel electrode (SCE) as reference electrode, and platinum wire electrode as counter electrode. 0.1 M PBS solution was used as supporting electrolytes in all electrochemical experiments. The electrochemical performances of 1 × 10^−5^ mol/L sunset yellow on various modified electrodes were investigated by cyclic voltammetry (CV) and second-derivative linear sweep voltammetry (SDLSV), with the potential scanning range of 0.4 ~ 1.2 V. Prior to all electrochemical measurements, an accumulation was performed under stirring at 500 rpm to improve the sensitivity. After 5 s rest, the CV or SDLSV were recorded at a scan rate of 100 mV/s, except where stated otherwise. All of the electrochemical tests were carried on the electrochemical workstation (CHI 760E, Shanghai Chenhua Inc., Shanghai, China). 

### 3.6. Analysis of Real Samples

Carbonated drinks, orange juice, and candy samples were purchased from a local supermarket. The same amount of candies was taken out from five packages and carefully grounded into fine powder. Then, the candies powder (about 1.0 g) was accurately weighed and dispersed in 10 mL deionized water under sonication for 1 h. Subsequently, the mixture was centrifuged at 4000 rpm for 10 min to remove insoluble substances. The 0.5 mL as-obtained supernatant was diluted to 10 mL with 0.1 mol/L PBS solution. The liquid samples (carbonated drinks and orange juice) were added into beaker and ultrasonicated for degasification. Then, 1.0 mL of the liquid samples was diluted to 10 mL with 0.1 mol/L PBS solution. Prior to electrochemical detection, accumulation step was performed in the sample solution to enhance the electrochemical response. Then, these sample solutions were detected by second-derivative linear sweep voltammetry while using our proposed Cu_2_O-ErGO/GCE. After each test, Cu_2_O-ErGO/GCE was scanned by cyclic voltammetry a 0.1 mol/L blank PBS solution for serval cycles to remove any adsorbents. The modified electrode can reused only when the response peak disappeared in the blank PBS solution. 

## 4. Conclusions

In this study, a promising electrochemical sensor based on the Cu_2_O-ErGO/GCE was developed toward sensing sunset yellow. The Cu_2_O-ErGO nanocomposites not only possess the advantages from individual component materials, but also exhibit obvious synergistic enhancement effects toward sunset yellow. The anodic peak current of sunset yellow on the Cu_2_O-ErGO/GCE increases by about 25 times as compared to that on the bare GCE. The proposed Cu_2_O-ErGO/GCE exhibits two linear regions, namely 2.0 × 10^−8^ mol/L–2.0 × 10^−5^ mol/L and 2.0 × 10^−5^ mol/L–1.0 × 10^−4^ mol/L, and the limit of detection is 6.0 × 10^−9^ mol/L (S/N = 3). The sensing performances in terms of linear response ranges and detection limit are comparable to, and even exceed the most reported modified electrodes, such as precious metal-based modified electrodes. Obviously, the Cu_2_O-ErGO/GCE have outstanding advantages over precious metal-based modified electrodes in term of the cost. Moreover, the response current is basically not affected by potential interfering compounds, suggesting the Cu_2_O-ErGO shows good selectivity. Besides, the good reproducibility was also obtained on the Cu_2_O-ErGO/GCE. Finally, Cu_2_O-ErGO/GCE have been successfully used for the quantitative detection of sunset yellow in real samples (i.e., carbonated beverage, orange juice, and candies) while using second-derivative linear sweep voltammetry. The satisfactory results are obtained with recovery rate is 98.75 ~ 102.5% and RSD is less than 2.85%. When compared with conventional analytical techniques (Table 4), the proposed method does not require expensive equipment, and time-consuming and complicated pretreatment procedures. Considering the considerable merits including low cost, rapid response, high sensitivity, as well as good selectivity and good reproducibility, the Cu_2_O-ErGO/GCE will have broad application prospects in the detection of sunset yellow in diverse beverages, foods, and nutrients.

## Figures and Tables

**Table 1:** Comparison the sensing performances toward the detection of sunset yellow between the existing modified electrodes and the proposed Cu_2_O-ErGO/GCE.

Modified Electrodes	Method	Metrological Parameters	Linear Range (μmol/L)	LOD (μmol/L)	Reference
PLPA/GCE	DPV	0.1 M phosphate-citrate buffer solution (pH 7.0); accumulation for 60 s	0.04–14	0.040	[42]
MIP/f-MWCNTs/GCE	DPV	0.1 M CBS solution (pH 5.0); accumulation for 30 min; scanned at 10 mV/s	0.05–100	0.005	[43]
Au-Pd-RGO/GCE	DPV	0.1 M PBS (pH 4.0); scanned at 50 mV/s	0.69–332	0.0015	[17]
CTAB-Gr-Pt/GCE	DPV	0.1 M PBS (pH 3.0); accumulation for 3 min	0.0085–1.0; 1.0–30	0.0042	[44]
GO/AgNPs-MIPs/GCE	LSV	0.1 M PBS (pH 5.5); accumulation for 7 min; scanned at 50 mV/s	0.1–0.6; 0.6–12	0.02	[45]
ILRGO-Au/GCE	SWV	0.1 M BR buffer solution (pH 7.0); accumulation for 300 s	0.004–1.0	0.00052	[13]
Au NPs/CPE	DPV	0.1 M PBS (pH 4.0); accumulation for 1 min; modulation amplitude = 60 mV and scan rate = 60 mV/s	0.1–2.0	0.03	[7]
MWCNT/GCE	DPV	0.1 M PBS (pH 7.0); accumulation at open circuit potential for 2 min; potential increment of 0.004 V, pulse amplitude of 0.05 V, and pulse period of 0.2 s	0.55–7.0	0.12	[46]
PDDA-Gr-Pd/GCE	DPV	0.1 M PBS (pH 3.0); accumulation for 5 min	0.01–10	0.002	[47]
Fe_3_O_4_@SiO_2_-NPs@MIP/Gr/GCE	DPV	0.1 M PBS (pH 8.0); pulse amplitude = 0.05 V; pulse interval time = 0.05 s, and scan rate = 0.02 V/s for differential pulse voltammetry	0.02–20	0.0055	[48]
Cu_2_O-ErGO/GCE	SDLSV	0.1 M PBS (pH 3.8); accumulation at 0.4 V for 180 s; scanned at 100 mV/s	0.02–20; 20–100	0.006	This work

**Table 2:** The reproducibility of Cu_2_O-ErGO/GCE for detection of sunset yellow.

No.	1	2	3	4	5	6	7
*i*_pa_ (μA)	21.07	22.34	21.36	22.04	22.29	22.85	22.24
Average value (μA)	22.02						
RSD (%)	2.78						

**Table 3:** The results of determination of sunset yellow in soft drink and candies (*n* = 3).

Samples	Original (μmol/L)	Added (μmol/L)	RSD (%)	Found (μmol/L)	Recovery (%)	RSD (%)
Sodas	ND ^1^	4	1.28	4.06	102.0	1.50
Orange juice	0.085	0.080	2.46	0.082	102.5	2.31
Candies	0.162	0.160	3.25	0.158	98.75	2.85

^1^ ND: Not detected.

**Table 4:** Advantages and drawbacks of previous reported protocols and our proposed protocol.

Protocols	Advantages	Drawbacks
Liquid chromatography	Reliable; good repeatability; high sensitivity; low LOD	Limited separation ability; Time-consuming; expensive equipment
Thin layer chromatography	Low cost apparatus	Organic solvents are often used; they have intensive disagreeable smell and cancerogenic activity
Spectrophotometry	Simultaneous identification and quantification; simple technique	Low sensitivity; extraction separation is needed for detection of dyes in complex product composition
Capillary electrophoresis	High column efficiency, short analysis time and minimal amounts of samples	Limited sensitivity & selectivity; severe matrix interferences
Electrochemical analysis (This work)	Low cost, rapid response, facile operate, high sensitivity and good selectivity	Portability needs to be improved; not disposable
